# Cardiomyopathy in Offspring of Pregestational Diabetic Mouse Pregnancy

**DOI:** 10.1155/2014/624939

**Published:** 2014-06-26

**Authors:** Daniel Dowling, Niamh Corrigan, Stephen Horgan, Chris J. Watson, John Baugh, Paul Downey, Fionnuala M. McAuliffe

**Affiliations:** ^1^UCD Obstetrics & Gynaecology, School of Medicine and Medical Science, National Maternity Hospital, University College Dublin, Dublin 2, Ireland; ^2^UCD Conway Institute of Biomolecular and Biomedical Research, University College Dublin, Belfield, Dublin 4, Ireland; ^3^Pathology, National Maternity Hospital, Dublin 2, Ireland

## Abstract

*Purpose*. To investigate cardiomyopathy in offspring in a mouse model of pregestational type 1 diabetic pregnancy. *Methods*. Pregestational diabetes was induced with STZ administration in female C57BL6/J mice that were subsequently mated with healthy C57BL6/J males. Offspring were sacrificed at embryonic day 18.5 and 6-week adolescent and 12-week adult stages. The size and number of cardiomyocyte nuclei and also the extent of collagen deposition within the hearts of diabetic and control offspring were assessed following cardiac tissue staining with either haematoxylin and eosin or Picrosirius red and subsequently quantified using automated digital image analysis. *Results*. Offspring from diabetic mice at embryonic day 18.5 had a significantly higher number of cardiomyocyte nuclei present compared to controls. These nuclei were also significantly smaller than controls. Collagen deposition was shown to be significantly increased in the hearts of diabetic offspring at the same age. No significant differences were found between the groups at 6 and 12 weeks. *Conclusions*. Our results from offspring of type 1 diabetic mice show increased myocardial collagen deposition in late gestation and have increased myocardial nuclear counts (hyperplasia) as opposed to increased myocardial nuclear size (hypertrophy) in late gestation. These changes normalize postpartum after removal from the maternal intrauterine environment.

## 1. Introduction

Diabetes mellitus is a common complication in pregnancy and is estimated to affect 2–5% of pregnant women [1]. Preexisting type 1 diabetes and preexisting type 2 diabetes account for 0.27% and 0.1% of births, respectively, and these figures are increasing globally [1]. The majority of pregnancies complicated by diabetes are a result of gestational diabetes (87.5%); the remaining 7.5% and 5% are due to pregestational type 1 and type 2 diabetes, respectively. Pregestational type 1 and type 2 diabetes are more serious and are responsible for greater adverse pregnancy outcomes [1, 2].

Parallel to the increasing global incidence of diabetes, the number of childbearing females presenting with pregestational diabetes is increasing. Maternal pregestational diabetes represents an arduous environment for embryonic and fetal development. Indeed, despite the improved obstetric care and better management of maternal hyperglycaemia over the last few decades, perinatal mortality and congenital abnormality rates still remain several-fold higher in pregnancies complicated by diabetes than in the background population [1–4]. Unlike gestational diabetes which occurs during midgestation, the stressors associated with pregestational diabetes are present during the first, most sensitive periods of fetal development, including the crucial period of organogenesis in the first trimester. An adaptive response by the fetus may result in and could contribute to modification of key metabolic processes and developmental organization, resulting in congenital defects and altered growth trajectories.

Among the numerous congenital malformations that have been associated with maternal pregestational diabetes, cardiovascular defects are the most frequent [5]. In a large prospective study of 192,618 live births in the UK, cardiovascular malformations were confirmed in 3.6% of babies born to pregestational diabetic mothers compared to 0.74% of babies born to nondiabetic mothers. This represented a fivefold increase in the risk of cardiovascular malformations in offspring of pregestational diabetic mothers [6]. Among the variety of cardiovascular malformations that can arise in these offspring hypertrophic cardiomyopathy represents a very common outcome. It has been shown to affect up to 75% of foetuses* in utero* and 40% of infants of pregestational diabetic mothers and can cause clinical symptoms in 5% [7–11]. Ullmo et al. described the diabetic foetal heart as being threatened in a double fashion [12]. Firstly, during the first trimester diabetes has a teratogenic effect on cardiogenesis by impairing the correct expression of genes coding for cardiac development [12–14]. Secondly, at the end of the second trimester or beginning of the third trimester, the fetus may be affected by hypertrophic cardiomyopathy. It has been shown that normally formed stillborn infants of diabetic mothers, when compared with appropriately grown stillborn nondiabetic infants, had heavier hearts and thicker ventricular free wall measurements when adjusted for birth weight [15]. Additionally there is data to suggest that offspring of pregestational diabetes are at an increased risk of cardiovascular disease later in life [16–18].

The aim of this study is to investigate mechanisms of offspring cardiomyopathy in pregestational type 1 diabetic pregnancy in a mouse model.

## 2. Materials and Methods

### 2.1. Generating a Mouse Model of Type 1 Diabetes in Pregnancy

Institutional ethical approval and Irish government licensing were obtained for all procedures. For all experiments the “Principles of laboratory animal care” (NIH publication number 85-23, revised 1985) were followed throughout. Type 1 diabetes was induced in female C57BL6/J mice using intraperitoneal streptozotocin (STZ) injections. A total of 54 female mice received STZ injections; these were divided into 6 groups and STZ injections were given to mice in each group on 6 different occasions. The first group (*n* = 10) received 50 mg/kg STZ over 5 consecutive days, with group 2 receiving a dosage regime of 75 mg/kg over 3 consecutive days. As there was a similar response to both STZ dosing regimes, we continued with the latter regime which resulted in fewer injections and less stress for the animals. Forty-four females received an STZ dose of 75 mg/kg/3 days. Diabetes was confirmed in female mice before pregnancy by taking fasting glucose measurements over two days. Diabetes was diagnosed when a fasting glucose was >11 mmol/L on two separate days. Blood was obtained via tail venipuncture and measured using an Accu-chek compact plus system (Hoffmann-La Roche Ltd. http://www.accu-chek.com/us/). Female C57BL6/J mice were mated with nondiabetic male C57BL6/J mice. The day of detection of a vaginal plug was designated E0.5 (embryonic day 0.5) when females were placed in an individual cage for the rest of the pregnancy. Healthy nondiabetic female C57BL6/J mice mated with nondiabetic C57BL6/J males acted as a control group. Mean fasting glucose prepregnancy in diabetic mice compared to controls was 13.5 ± 1.0 versus 5.4 ± 0.44 mmol/L, and towards the end of pregnancy (E16.5) it was 21.7 mmol/L ± 6.3 versus 5.3 ± 0.44 mmol/L.

### 2.2. Histology

In order to obtain histological data from embryonic hearts, pregnant animals were sacrificed at E18.5 using cervical dislocation while being under heavy sedation (3% isoflurane). Once death was confirmed the maternal abdomen was opened using 30° angled scissors. Embryos were removed from the uterus and placed on iced phosphate buffer saline (PBS). Embryos were removed from the individual yolk sac using iridectomy scissors and hearts from the 6- and 12-week-old mice were excised. All samples were fixed in 10% formalin for a minimum of 24 hours, processed, and subsequently paraffin-embedded. Five *μ*m sections were cut using a microtome and mounted onto poly-l-lysine-coated glass slides. Samples were stained with either haematoxylin and eosin (H&E) or Picrosirius red using a Leica autostainer XL. The nuclear stain haematoxylin enables measurement of the number and size of nuclei. Picrosirius red detects collagen isoforms 1 and 3, which reflects the presence and extent of any abnormal collagen deposition in the offspring hearts. The Picrosirius red staining procedure involved both automated (Leica autostainer XL) and manual steps. Sections were deparaffinised and hydrated in the Leica autostainer and then placed in 5% phosphomolybdic acid (PMA). Sections were then rinsed in distilled water before being placed in picrosirius red (1 : 1000 dilution; Direct red 80 (25%) in picric acid). Sections were then transitioned from 0.4% hydrochloric acid (HCL) to 70% ethanol before dehydration in the Leica autostainer.

### 2.3. Aperio ScanScope XT High Throughput Scanning System

**Figure 1:**
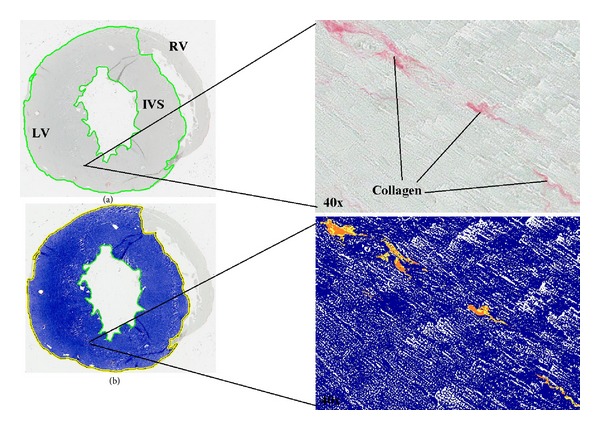
Histological images of six-week-old offspring of type 1 diabetic mouse demonstrating the application of the Aperio software. (a) Full image taken with Aperio digital scanning microscope. LV: left ventricle wall; RV: right ventricle wall; IVS: interventricular septum. (b) Mark-up image showing the detection of collagen by the Aperio positive pixel count algorithm. Blue represents negative background and yellow/orange represents positive collagen detection, as shown in corresponding 40x magnified images.

The Aperio ScanScope XT system (http://www.aperio.com) is capable of scanning glass slides at 20x or 40x magnification to create seamless, true-colour digital slide images. The main advantage of this type of image analysis is the inherent objectivity which excludes potential errors associated with manual scoring and analysis; it also provides the facility to perform batch analysis and the use of associated analysis software (Spectrum). Spectrum tools allow the user to annotate, extract, highlight, and analyse the whole slides or specific regions with the use of a comprehensive image analysis algorithm library. To investigate the possible presence of cardiomyocyte hyperplasia and/or hypertrophy, a nuclear algorithm was modified and applied to the scanned H&E stained slides. The algorithm calculated two main parameters, (1) the number of myocyte nuclei and (2) the size of myocyte nuclei. Following optimisation with the algorithm settings on the Aperio software, the following size exclusion settings were selected to ensure only myocyte nuclei were assessed: minimum nuclear size, 10 *μ*m^2^; minimum nuclear size, 162 pixels; minimum compactness, 0.22; minimum elongation, 0.25. To investigate the extent of collagen deposition in offspring hearts, Picrosirius red staining was used. A positive pixel count algorithm was used to automatically quantify the area occupied by the dark pink stain colors representing collagen within each scanned slide image (Figure 1(a)). Calibration of individual staining patterns was performed by specifying the requisite colour (range of hues and saturation) and limits for the desired intensity range. Required input parameters for each stain were based on the HSI (hue, saturation, and intensity) colour model. To detect the dark pink colour of collagen with Picrosirius red, a hue value of 0.8 was specified. The hue width value of 0.5 was used to allow inclusion of a moderate range of colour shades. A collagen volume fraction was calculated based on the percent of dark pink collagen staining quantified within a tissue section.

### 2.4. Statistical Analysis

The SPSS 18 statistics package was used to perform statistical analysis. The data were normally distributed and independent two-tailed equal variance *t*-tests were used when comparing the two groups. A *P* value of <0.05 was considered to be significant.

## 3. Results

### 3.1. Cardiomyocyte Nuclei Size

**Figure 2:**
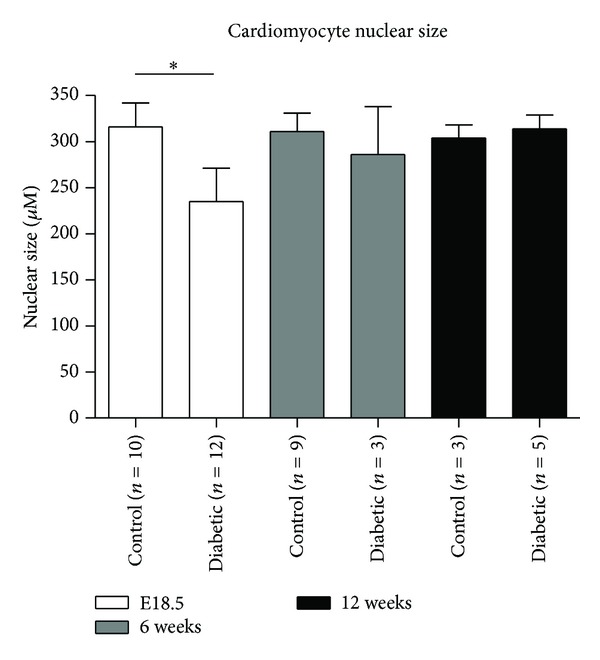
Comparison of cardiomyocytes nuclear size. Cardiac tissues derived from offspring of control and diabetic groups from E18.5 (white bars), 6 weeks (grey bars), and 12 weeks (bars) of age were stained with haematoxylin and eosin. Slides were digitally scanned and the nuclear size of the cardiomyocytes was calculated using a modified algorithm from Aperio. Mean nuclear area (*μ*M) is presented. *P < 0.05.

Analysis with the nuclear algorithm shows that offspring of diabetic mothers have significantly smaller nuclei (defined by pixilation) than the control mice at embryonic day 18.5 (235 ± 125 versus 316 ± 82; *P* < 0.05). The same difference was not found at 6 and 12 weeks of age (Figure 2). Furthermore, when all age groups were pooled into their relevant groups (diabetic or control), a significant difference was still present. Diabetic nuclei were significantly smaller than control nuclei (272 ± 101 versus 312 ± 66; *P* < 0.05) (Figure 2).

### 3.2. Cardiomyocyte Nuclear Number

**Figure 3:**
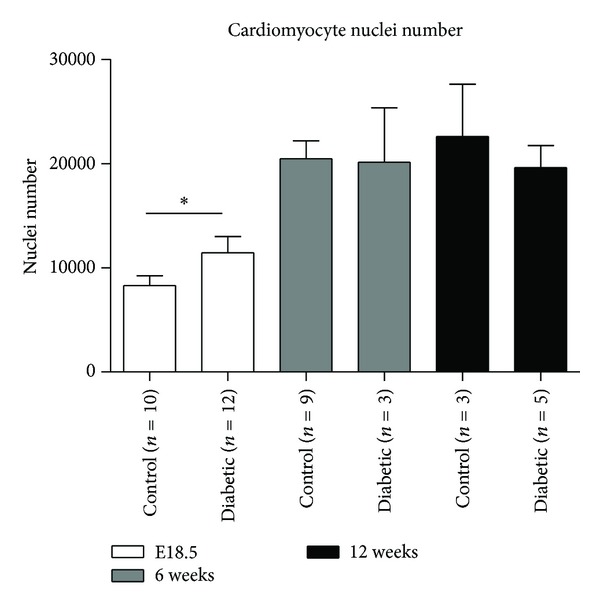
Comparison of cardiomyocytes nuclear number. Cardiac tissues derived from offspring of control and diabetic groups from E18.5 (white bars), 6 weeks (grey bars), and 12 weeks (bars) of age were stained with haematoxylin and eosin. Slides were digitally scanned and the number of nuclei of the cardiomyocytes was calculated using a modified algorithm from Aperio. Mean nuclear number is presented. *P < 0.05.

There was a significant increase in cardiomyocyte nuclei number in diabetic offspring at embryonic day 18.5 (E18.5) compared to controls (11440 ± 5401 versus 8279 ± 3058; *P* < 0.05). No significant difference was found between the groups at 6 and 12 weeks of age (Figure 3).

When analysing the relationships between nuclear features and echocardiography parameters, significant correlations were only observed when combining data from the 6- and 12-week groups. A positive correlation between increasing nuclei number and increasing interventricular septal thickness (IVS) was observed (Pearson correlation: 0.598∗;* R* ^2^ linear: 0.358; *P* < 0.05).

### 3.3. Collagen Deposition

**Figure 4:**
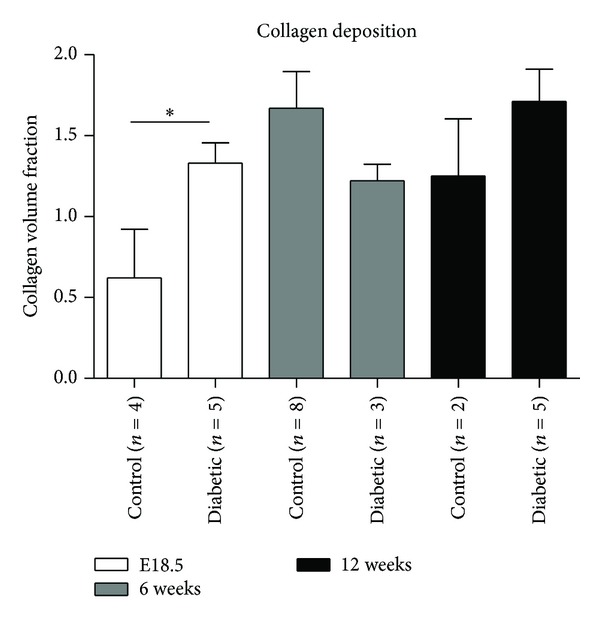
Comparison of the degree of collagen deposition. Cardiac tissues derived from offspring of control and diabetic groups from E18.5 (white bars), 6 weeks (grey bars), and 12 weeks (bars) of age were stained with Picrosirius red to assess the extent of collagen deposition. Slides were digitally scanned and a positive pixel count algorithm was used to automatically quantify the area occupied by the dark pink stain colors representing collagen. The collagen volume fraction was calculated and is presented. *P < 0.05.

Histological analysis of collagen volume fraction revealed that there was a significant increase in collagen deposition present in the myocardium of diabetic offspring at embryonic day 18.5 (E18.5) compared to controls (1.33 ± 0.28 versus 0.62 ± 0.60; *P* < 0.05). This difference was not present at 6 and 12 weeks of age (Figure 4).

## 4. Discussion

This study shows that myocardial samples from offspring of pregestational type 1 diabetic pregnancies display nuclear characteristics of myocyte hyperplasia as opposed to hypertrophy. Cardiac myocyte nuclei were smaller and more numerous in late gestation (embryonic day 18.5) compared to control offspring. The results also show an increase in collagen deposition in the hearts of offspring of pregestational type 1 diabetic pregnancy in late gestation (embryonic day 18.5). These alterations in cardiomyocyte nuclei size and number and collagen deposition were shown to normalize and return to control levels by 6 and 12 weeks, representing adolescence and adulthood, respectively.

Hyperplasia is the main mechanism involved in cardiac development* in utero*, soon after birth hypertrophy takes over as the main mechanism by which cardiac mass increases. This increase in cardiac mass is a characteristic of hypertrophic cardiomyopathy and is mediated by an increase in the total mass of the myocytes and in the total mass of interstitial fibrosis. Previous studies have shown that embryos and neonates from diabetic pregnancy experience enlargement of the heart and postulated that this was due to a combination of hypertrophy and hyperplasia of the cardiomyocytes and fibroblasts [12, 15, 19]. Our results show increased numbers of cardiac myocyte nuclei and increased collagen deposition in the offspring of pregestational diabetics. These findings support the hypothesis that hyperplasia is the main mechanism involved in the increase in cardiac mass in late gestation and cardiomyocyte hypertrophy plays a less significant role in the development of foetal hypertrophic cardiomyopathy. In addition, correlations with echocardiography data highlight a significant positive correlation between nuclei number and interventricular septal thickness. This result further suggests that there may be a link between cardiomyocyte number and hypertrophic cardiomyopathy, possibly inferring a role for increased hyperplasia in hearts of offspring from type 1 diabetic mothers, although further studies would be required to confirm this hypothesis.

Studies have shown that the diabetic intrauterine environment is characterised by increased reactive oxygen species [20–22], and these have been shown to directly impair contractile function [23] and affect myocardial development by means of activating many downstream signalling pathways involved in myocardial growth, matrix remodelling, and cellular dysfunction [20, 22]. ROS have also been shown to activate matrix metalloproteinases and embryonic fibroblasts, both of which lead to increased collagen and fibronectin deposition [22, 24]. In mid-to-late gestation embryos begin producing their own insulin to regulate glycaemia and growth. Unfortunately this process quickly changes from a helpful mechanism needed for ameliorating high levels of circulating blood glucose to one of a pathogenic accomplice to hyperglycemia. High levels of foetal insulin are produced in response to high levels of circulating blood glucose. This results in rapid foetal growth and offspring born large for gestational age, known as macrosomia. Foetal hyperinsulinemia has also been associated with the development of hypertrophic cardiomyopathy [25, 26]. Insulin has been shown to stimulate fibroblast proliferation [27], which again may lead to increased collagen deposition. However, the activation of cardiac fibroblasts can have a direct effect on the number of cardiomyocytes. Ieda et al. found that embryonic cardiac fibroblasts induced the proliferation of cardiomyocytes [28]. They identified fibronectin, collagen, and heparin-binding EGF-like growth factor as embryonic cardiac fibroblast specific signals that collaboratively promoted cardiomyocyte proliferation through *β*1 integrin signalling in a paracrine fashion [28]. It has been shown that normally formed stillborn infants of diabetic mothers, when compared with appropriately grown stillborn nondiabetic infants, had heavier hearts and thicker ventricular free wall measurements when adjusted for birth weight [15]. Biochemical markers of cardiac dysfunction (Pro-B-type natriuretic peptide and Troponin-T) have also been shown to be elevated in infants of diabetic mothers, especially those with cardiomyopathy and poor perinatal outcome [29].

Our group previously identified global cardiac hypertrophy in late gestation (embryonic day 18.5) of diabetic pregnancy with ultrasound [11, 15, 30]. Increased interventricular septal and posterior wall thickness was observed in late pregnancy of pregestational diabetic mothers. We have shown in this present study that the structural alterations found in embryos of late stage diabetic pregnancy may be due to excessive cardiomyocyte hyperplasia and increased collagen deposition resulting from cardiac fibroblast cell activity. These processes may be due to the pregestational diabetic intrauterine environment characterized by hyperglycemia, increased reactive oxygen species production, and foetal hyperinsulinemia.

Although previous studies have postulated that cardiomyocyte hypertrophy and hyperplasia in addition to fibrosis were the means by which cardiac mass increases; to our knowledge this is the first study to show that it is cardiomyocyte hyperplasia that plays a much more central role in this pathogenesis as opposed to hypertrophy. This is in addition to increased collagen deposition. This study provides a novel insight into the cardiac changes that may occur in the embryos of a mouse model of pregestational type 1 diabetic pregnancy. One limitation to the study is the relatively low numbers of subjects available for examination, as cohorts of animals were sacrificed for different experimental procedures. Future research may benefit from larger numbers to improve statistical power and also investigate cardiac fibroblast activity, as these cells have shown to be important players in the development of cardiomyopathies.

## 5. Conclusion

This data suggests that pregestational type 1 diabetes impacts offspring cardiomyocyte development resulting in increased cardiomyocyte proliferation and increased cardiac fibroblast activity. These changes appear to resolve by adolescence but may contribute to an increased risk of cardiovascular disease in later life.
